# Case Report: Presence of Anti-MAG in the CSF Can Be Associated With a Neurodegenerative Process With Frontal Involvement

**DOI:** 10.3389/fneur.2022.847798

**Published:** 2022-05-25

**Authors:** Guillaume Dorcet, Marie Benaiteau, Fabienne Ory-Magne, Antoine Blancher, Jérémie Pariente, Françoise Fortenfant, Chloé Bost

**Affiliations:** ^1^Département de Neurologie, Hôpital Pierre Paul Riquet, CHU de Toulouse, Toulouse, France; ^2^INSERM U1043 – CNRS UMR 5282, INFINITY, Toulouse, France; ^3^Laboratoire d'Immunologie, Institut Fédératif de Biologie, CHU de Toulouse, Toulouse, France; ^4^INSERM ToNIC, Toulouse NeuroImaging Center, Université de Toulouse, Université Paul Sabatier, Toulouse, France

**Keywords:** MAG, autoantibodies, neurodegenerative diseases, differential diagnosis, myelin alteration

## Abstract

**Background:**

Autoimmune encephalitis (AIE) is an increasingly broad nosological framework that may clinically mimic neurodegenerative diseases (NDDs).

**Cases Reported:**

We describe here the clinical, radiological, electrophysiological, and biological evolution of three patients. Two women aged 73 and 72 years and a 69-year-old man presented with complex cognitive and focal neurological symptoms and each had a predominant frontal dysexecutive involvement and an unexpectedly high titer of anti-MAG antibodies in the serum and cerebrospinal fluid (CSF). The question of an autoimmune cause was raised. After 2 years of follow-up and, for two of them, without improvement despite immunosuppressive treatments, diagnoses of NDD were eventually retained: post-radiation encephalopathy, progressive supranuclear palsy (PSP), and Alzheimer's disease.

**Conclusion:**

The presence of a high titer of anti-MAG antibodies may be found in NDD. It could reflect cerebral tissue damages, particularly in the case of significant frontal involvement. Atypical presentations may lead to a search for a paraneoplastic neurologic syndrome or AIE. However, the indirect immunofluorescence staining positivity on a monkey cerebellum section linked with anti-MAG antibodies should not lead to those diagnoses being retained.

## Introduction

In the expanding field of autoimmune encephalitis (AIE), some dysimmune processes involving the central nervous system can mimic neurodegenerative diseases (NDDs), such as Parkinson's disease (PD) (1), progressive supranuclear palsy (PSP) (2), Huntington's disease (HD) (3), Creutzfeldt-Jakob's disease (CJD) (4), or Alzheimer's disease (AD) (5). The description of new cases of AIE mimicking NDD contributes to broadening the AIE spectrum, especially when movement disorders are present, making differential diagnosis more difficult. Indeed, numerous antibodies directed against antigenic targets present in the central nervous system (CNS), at the membrane or intracellular level, have been described in association with those NDD-like diseases, whether they are involved in the pathophysiology through a direct role on their target or not (6).

We report here three patients presenting central neurological involvement with abnormalities that led to a search for arguments in favor of AIE. In these three patients, the initial presentation and the biological data showing atypical immunological marking and the presence of anti-MAG IgM antibodies in the cerebrospinal fluid (CSF) could have led to the suspicion of autoimmune involvement, strong enough to lead to the establishment of immunosuppressive treatment for one of them. A diagnosis of NDD associated with the satellite presence of a high titer of anti-MAG antibodies was finally retained.

## Cases Reported

The three patients were examined at the Toulouse University Hospital between 2016 and 2020, with a standardized clinical evaluation, neuropsychological assessment, and biological and imaging tests. All cases were discussed in multidisciplinary concertation meetings including neurologists, immunologists, and neuroradiologists.

### Case Number 1

A 73-year-old woman was treated in 2015 for B cell acute lymphoblastic leukemia with multidrug therapy according to the “EWAL plus rituximab” protocol, combined with 10 sessions of prophylactic encephalic radiotherapy, followed by aracytin, intrathecal methotrexate, rituximab, and dexamethasone in maintenance. Neurological symptoms appeared in 2017, during the last year of consolidation, with hypokinetic walking with early falls, asthenia, apraxia, and progressive, anterograde amnesia, complicated within 2 years by left-predominant pyramidal syndrome, kinetic cerebellar syndrome, and cognitive impairment with clear predominance of executive functions (Frontal Assessment Battery [FAB] at 3/18 for an MMSE score of 20/30).

**Figure 1:**
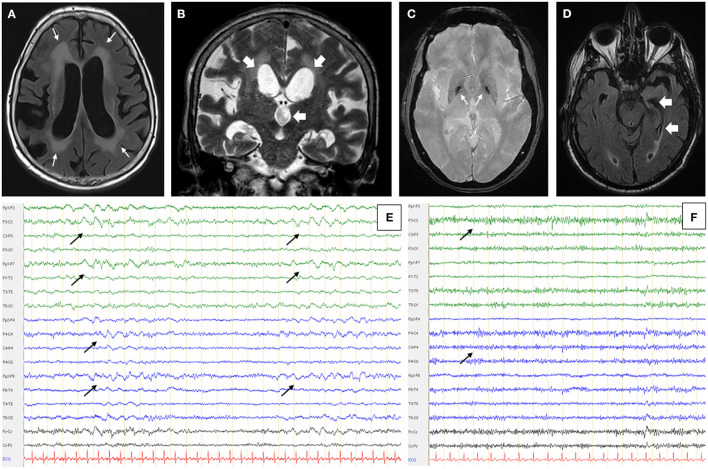
Patient's MRI and EEG. Patient N°1 brain MRI in axial FLAIR (fluid attenuated inversion recovering) sequence **(A)** and coronal T1 weighted sequence **(B)** showed periventricular confluent leukopathy (thin white arrows - A), cortical and subcortical atrophy predominant on the right hemisphere and a significant dilatation of lateral and third ventricles (large white arrows - B). **(C)** Patient N°2 had a hyposignal of the two pallidal tips in axial T2* sequence (white arrows). **(D)** Hippocampal atrophy, predominant on the left side, on patient's N°3 axial FLAIR MRI (white arrows). All MRI were performed on 3 Tesla devices (Magnetom Skyra, Siemens Healthcare, Erlangen, Germany and Achieva 3.0 T, Philips, Amsterdam, Netherlands). **(E)** Patient N°1 also had recurrent bilateral anterior delta waves on a 6.5 Hz basic rhythm (black arrows). **(F)** Patient N°2 had a non-pharmacological, bilateral and anterior fast rhythm at 14 Hz (black arrows). EEGs were performed and read on Deltamed devices and software (Natus Neurology, Pleasanton, USA).

Brain MRI showed diffuse cortico-subcortical atrophy, periventricular and brainstem leukopathy, and ventricular dilatation consistent with normal pressure hydrocephalus (NPH), secondarily confirmed by hydrodynamic testing (Figures 1A,B). EEG showed theta slowing with the presence of numerous anterior delta waves (Figure 1E). Biological tests revealed a monoclonal IgM kappa gammopathy (2.5 g/L) with an elevated alpha-fetoprotein, linked to a heterozygous profile without ataxia-telangiectasia.

**Figure 2:**
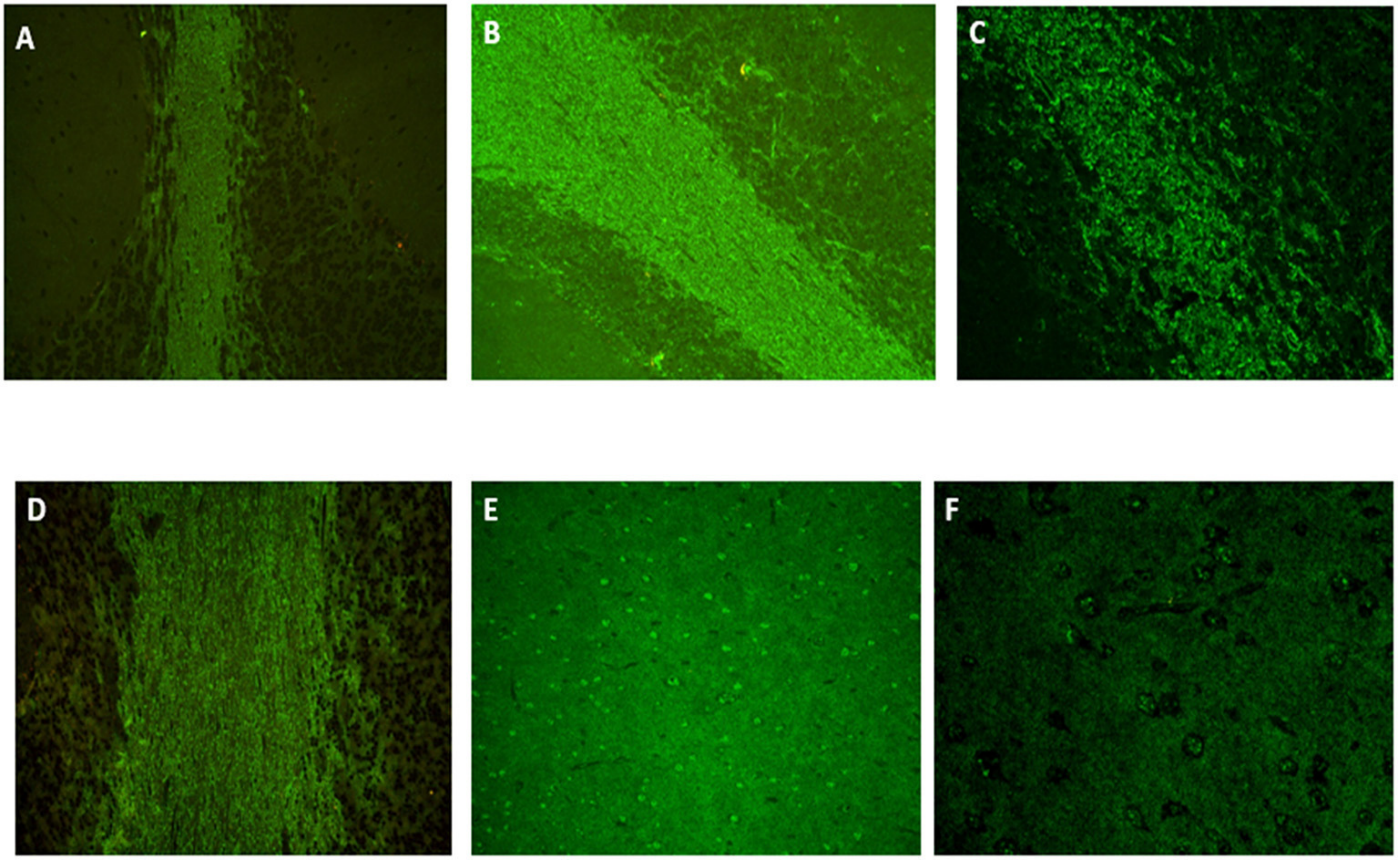
Indirect immunofluorescence on monkey cerebellum slices (ref. 0665042225.10, NOVA Lite, Inova, Falls Church, USA) showing atypical and intense staining of the white matter for patient N°1 [**(A)**, × 20, IgM kappa monoclonal gammopathy], identical when a secondary antibody anti-IgG, anti-IgM or anti-kappa was used but negative with an anti-lambda antibody. The same staining was found for patient N°2 with anti-IgG [**(B)**, × 20], anti-IgM and anti-lambda [**(C)**, × 40, IgM lambda monoclonal gammopathy] but negative with anti-kappa. Patient N°3 (IgG kappa monoclonal gammopathy) had a positive staining only with anti-IgG and anti-kappa secondary antibody [**(D)**, × 20]. A fine speckled staining was also present in cortex [**(E)**, × 20 and **(F)**, × 40] for all patients. Immunostaining positivity was only present within sera but negative within CSF. Anti-MAG antibodies presence was tested on monkey nerve slice (ref. 066504210.10, NOVA Lite) and confirmed by ELISA (ref. EK-MAG, Bhlmann, Bremen, Germany).

A search for anti-neuronal antibodies was carried out in order not to omit a paraneoplastic neurological syndrome. The presence of anti-neuronal auto-antibodies was tested by indirect immunofluorescence on monkey cerebellum and cerebrum slices (NOVA Lite), rat hippocampus slices (Euroimmun), and by identification tests; by immunodot for intracellular antigens (Eurobio Scientific) and transfected cells for the membrane target (Euroimmun), respectively. An atypical appearance of the serum with anti-IgM, anti-IgG, and anti-kappa only, staining the white matter (Figure 2A) on the monkey cerebellum slice led to further investigations. This pattern was found to be associated with the presence of anti-MAG IgM antibodies, found in a high titer in serum (32,772 IU/mL) and CSF (11,189 IU/mL, with a serum/CSF index=3 [serum anti-MAG (IU/ml): CSF anti-MAG (IU/ml) × 1,000]) while the intracellular and membrane anti-neuronal antibodies were negative. The barrier index ([CSF albumin (mg/ml): serum albumin (mg/ml)] × 1,000) was increased to 10.87 for a normal of <6.5 and there was no oligoclonal band restricted to the CSF; proteins and cell count were normal.

In view of the progressive evolution over several years and the history of the disease, particularly the onset under immunotherapy, the diagnosis of probable AIE according Graus et al. (5) criterion was ruled out and it was concluded that the patient had post-radiation encephalitis complicated by NPH. Unfortunately, the patient died from a postoperative subdural hematoma due to CSF shunt surgery.

### Case Number 2

A 74-year-old woman without significant medical history presented with dysphonia, dysarthria, word finding, walking ataxia, social disinhibition, anxiety, irritability, and inappropriate laughter appeared within few months. Two years later, in 2018, she developed kinetic and static cerebellar syndrome, pyramidal syndrome, left hemichorea, grasping reflex, and staring predominant in verticality. Cognitively, there was significant dysexecutive impairment (FAB 7/18) contrasting with preserved memory and instrumental functions (MMSE 25/30).

Brain MRI showed an unspecific vascular leukopathy and mild cortico-subcortical atrophy. There was also a trivial hypointense signal of the pallidal tips on the T2^*^-weighted sequence (Figure 1C). The EEG recovered a pattern marked by non-pharmacological anterior fast activity on an alpha background (Figure 1F). The ENMG (electroneuromyogram), carried out because of the clinical resemblance with the cohort of Zis et al. (7), was normal, apart from a banal bilateral carpal tunnel syndrome. Skin biopsy did not reveal any evidence of endovascular lymphoma.

Biologically, we found an IgM lambda and IgE lambda gammopathy (IgM titer at 4.29 g/L). Genetic testing for fragile X syndrome, Huntington's disease, and Niemann-Pick type C disease was negative. In the CSF, an isolated amyloidopathy (362 pg/mL, normal value superior to 500 pg/mL), without taupathy, high proteins, cells count, or oligoclonal band, was retrieved. The barrier index was slightly increased at 7.32. As part of the search for Whipple's disease (all tests negative), a gastroduodenal endoscopy was performed and found a well-differentiated neuroendocrine duodenal lesion treated by excision. Because of the rapidly progressive cerebellar involvement and atypical presentation, anti-neuronal antibodies were researched. The same atypical IgM, IgG, and lambda staining as in patient number 1 was revealed (Figures 2B,C,E,F) and further investigations revealed a high level of anti-MAG IgM in the serum (>70,000 IU/mL) and in the CSF (37,432 IU/mL), with a serum/CSF index > 1.9. Treatment associating rituximab (1 g on day 1 and day 15) and corticosteroids was initiated in the hypothesis of a probable AIE (5) with involvement of anti-MAG antibodies. There was no clinical improvement, and the subsequent evolution of the disease was that of type C progressive supranuclear palsy (PSP-C), the diagnosis was finally retained (8). The patient became bedridden and died at 78.

### Case Number 3

A 69-year-old man presented in 2016 with mixed aphasia, predominantly on expression, visual hallucinations, and Capgras syndrome. He had had a Gleason 7 prostate adenocarcinoma, surgically removed in 2017. The same year, he also presented with an extrapyramidal syndrome and cognitive deterioration including apraxia and anterograde verbal and visual amnesia. Again, executive functions were mainly affected (FAB 3/18 for MMSE 15/30).

Brain MRI showed a mild leukopathy, usual at that age, hippocampal atrophy, and a right-sided occipital meningioma (Figure 1D). The EEG was normal. Biological work-up showed an IgG kappa monoclonal gammopathy at 14.28 g/L and a bright fluorescence of the white matter (with anti-IgG and anti-kappa only) on monkey cerebellum slices when anti-neuronal antibodies were searched (Figure 2D). Again, the atypical staining was consistent with the presence of anti-MAG IgM positivity. The titer of anti-MAG in the serum was quite elevated (9,878 IU/mL) but below the range in CSF (<1,000 IU/mL, with a serum/CSF index >9.9). There was also a huge increase in the total (1,554 pg/mL for a norm below 450 pg/mL) and phosphorylated tau protein (195 pg/mL; norm below 60 pg/mL) and a decrease in Aβ_1−42_ protein (205 pg/mL for a norm greater than 500 pg/mL). The barrier index was slightly elevated at 7.97; proteins and cell count were normal, and the search for oligoclonal bands was negative. The infectious work-up in serum and CSF was negative.

A frontal variant of Alzheimer's disease, based on clinical presentation and tau and amyloid protein rates was retained and confirmed after 4 years of follow-up.

## Review of Literature

We report here three cases of various NDDs (post-radiation encephalopathy, PSP-C, and Alzheimer's disease) and the presence of a high titer of anti-MAG antibodies in serum and CSF without neuropathy.

The presence of anti-neural antibodies in the serum or CSF of patients with NDDs has been reported in the past. Anti-GlyR and anti-NMDAR antibodies have been demonstrated in the context of CJD, anti-GlyR antibodies with genetic dystonia, anti-NMDAR antibodies with MELAS syndrome (mitochondrial encephalopathy with lactic acidosis and stroke-like episodes), and anti-GABA_A_-R antibodies with HD (6). Beside, previous studies have already reported an elevation of anti-MAG antibodies in the serum of AD (9) and in typical or atypical Parkinsonian syndromes (10). Antibodies were here only satellites of the NDD, without apparent involvement in the disease process. However, a case of genuine AIE with anti-LGI1 antibodies has been reported, evaluated in parallel with CJD, raising the question of a causal link between the two pathologies (11).

An inflammatory myelin alteration process in the CNS is mainly described in the field of anti-MOG-associated diseases (12). The myelin oligodendrocyte glycoprotein (MOG) is only expressed at the outer layer of the myelin sheath and oligodendrocyte membranes, making it a potential target for immune responses. MAG (myelin-associated glycoprotein) is a protein present in the periaxonal space, between oligodendrocytes or Schwann cells and neurons, expressed in the central and peripheral nervous system, and is also a potential target for immune response in these two parts of the nervous system. However, anti-MAG antibodies are classically described as being associated with distal demyelinating neuropathy occurring in patients with monoclonal gammopathy (13).

The more frequent presence of autoantibodies directed against white matter epitopes in patients with NDD raises the question of an active participation of macroglial cells in these diseases (14, 15). A large, post-mortem, immunohistological study of 55 AD patients showed an elevation of the MAG/PLP ratio in AD-associated white matter lesions. The change in the ratio was mainly due to a decrease in the PLP rate (16). Then, the occurrence of anti-MAG autoantibodies could be interpreted as a marker of a mechanism to promote neuronal regrowth. Indeed, it is well-described that MAG is involved in the inhibition of axonal growth (17), and a study in a mouse model of stroke demonstrated that early administration of anti-MAG antibodies resulted in better recovery and less extension of the necrotic zone (18).

Interestingly, a recent study on an animal model of PD found an overexpression of MAG in frontal regions, confirmed by transcriptomic analysis of the autopsies of PD patients (19). Our patients share a particularly important impairment of executive functions, marked by collapsed FAB scores and various impairments involving cortical and subcortical frontal networks, such as social inhibition and oral expression. Two of them also had EEG abnormalities in the anterior regions. The occurrence of anti-MAG antibodies alongside NDD could then be a sign of significant involvement of anterior brain regions.

## Discussion

Even if a possible or probable AIE could be initially evoked, the involvement of a neuroinflammatory, immune-mediated process can likely be ruled out in the cases we present here. First, there was no evidence of intrathecal synthesis, although it was not excluded: no oligoclonal band, even if this element is just a fickle surrogate for neuroinflammation (20) and a higher level of anti-MAG in serum than in CSF for all patients. Then, patient number 1 developed neurologic disorders even if she had benefited shortly before from strong immunosuppressants and was still being treated with corticosteroids. Moreover, in the hypothesis of an autoimmune cerebellitis associated with anti-MAG antibodies, patient number 2 was treated with rituximab and corticosteroids without any effect, contrary to the few reported cases of AIE associated with anti-MAG antibodies (7). Also, these cases differed from those reported here in several aspects: they presented with cerebellitis and not diffuse encephalitis; four of the five patients described also had a polyneuropathy associated with the central involvement, and all of them had strictly IgM monoclonal gammopathy. For our three patients, while all had monoclonal gammopathy of unknown significance (MGUS), the nature of the heavy and light chains of the monoclonal peak differed from one patient to another.

Taken together, these data point to the hypothesis of the overexpression or abnormal exposure of a neural surface antigen in the context of NDD that favors the emergence of autoantibodies. Indeed, in addition to anti-MAG antibodies, the elevation of other antibodies directed against myelin (MOG, MBP, PLP) and, more broadly, glial (S100B) proteins has been described in several NDDs (9, 21). The HNK-1 epitope recognized by anti-MAG antibodies, not tested here, is also present on multiple glial glycoproteins in a constant (P0, PMP22) or transient (contactin, NCAM, F3, F11, AG-1) manner (22), which reinforces the hypothesis of antigenic exposure through the involvement of white matter during NDD. The presence of anti-MAG antibodies at a high titer in the CSF of our patients can be explained by the occurrence of a blood-brain barrier (BBB) disruption, demonstrated by an elevated barrier index shown for our three patients. The dysfunction of the BBB is part of the pathophysiological process of NDD (23). That may suggest a break in the immune privilege of the CNS, favoring a cross immune reaction against an epitope shared by many neural molecules. The occurrence of anti-MAG antibodies could be favored by the presence of an underlying monoclonal gammopathy. This is coherent with the fact that the light chain positivity on immunostaining corresponded to the patient's monoclonal gammopathy.

Here, the coincidental association of MGUS with neurodegenerative disease is unlikely, because it is unusual that MGUS is associated with CNS involvement rather than PNS (24). Plus, in our cases, immunostaining in the CSF corresponded to MGUS immunoglobulin. In addition, the frontal impairment in the foreground of the clinical presentation of these three patients was surprising.

In conclusion, we reported here three cases of complex encephalopathies with predominant frontal involvement, associated with a high titer of anti-MAG antibodies in serum and CSF, which raised the question of the differential diagnosis between NDD and AIE. It turned out that these patients had NDD with associated biological autoimmunity signs without obvious involvement of these antibodies in the development of symptoms. We would like to inform clinicians and immunologists of the possibility of high anti-MAG antibodies titers in the CSF, causing an atypical indirect immunofluorescence positivity on monkey cerebellum slices, a satellite of NDD.

## Data Availability Statement

The raw data supporting the conclusions of this article will be made available by the authors, without undue reservation.

## Ethics Statement

The studies involving human participants were reviewed and approved by the French Ethical Southwest and Overseas Committee (SOOM2) under the number DC20162804. Written informed consent for participation was not required for this study in accordance with the national legislation and the institutional requirements.

## Author Contributions

CB and FF contributed to conception and design of the study. GD organized the database and wrote the first draft of the manuscript. GD and CB performed the statistical analysis. MB, FO-M, AB, JP, FF, and CB wrote sections of the manuscript. All authors contributed to manuscript revision, read, and approved the submitted version.

## Funding

This work was funded by the University Hospital of Toulouse, France (DSL-R21132BB).

## Conflict of Interest

The authors declare that the research was conducted in the absence of any commercial or financial relationships that could be construed as a potential conflict of interest.

## Publisher's Note

All claims expressed in this article are solely those of the authors and do not necessarily represent those of their affiliated organizations, or those of the publisher, the editors and the reviewers. Any product that may be evaluated in this article, or claim that may be made by its manufacturer, is not guaranteed or endorsed by the publisher.
